# Modified Leukocyte Filter Removes Tumor Cells from the Salvaged Blood

**DOI:** 10.1371/journal.pone.0130864

**Published:** 2015-06-22

**Authors:** Kai Mei, Lei Du, Min Yan, Zhaohui Zhang, Fengjiang Zhang, Lina Gong, Kai Sun, Jie Zhang, Yumin Tang, Chunling Jiang, Jin Liu

**Affiliations:** 1 Department of Medical Oncology, Sichuan Cancer Hospital, Chengdu, Sichuan, China; 2 Department of Anesthesiology and Translational Neuroscience Centre, West China Hospital, Chengdu, Sichuan, China; 3 Department of Anesthesiology, Affiliated No. 2 Hospital, Zhejiang University, Hangzhou, China; 4 Department of Anesthesiology, Sichuan Cancer Hospital, Chengdu, Sichuan, China; 5 Key Laboratory of Transplant Engineering and Immunology, Ministry of Health and West China Hospital, Sichuan, China; The Ohio State University, UNITED STATES

## Abstract

**Background:**

Intraoperative blood salvage, an effective blood conservation strategy, has not been applied in onco-surgery, because of potential malignant cell contamination. In this study we tested effectiveness of a modified leukocyte depletion filter (M-LDF) for removal of tumor cells.

**Materials and Methods:**

The effects of M-LDF and regular LDF on removal of cells (HepG2 cell line) were compared. The safety of M-LDF was tested with blood (collected and washed during onco-surgery), the salvaged blood mixed with tumor cells from the solid tumor of the same patient, or mixed with HepG2 cells (n=30 in each protocol). Cancer cells were identified by flow cytometry, culture and bioassay with and without filtration.

**Results:**

M-LDF removed 5-log of HepG2 and nucleated cells, which was much higher than regular LDF, and cells were destroyed when they passed through M-LDF. Cytokeratin-positive cells in all samples were removed by M-LDF. Invasive growth adherent cells were found in most of unfiltered samples and 67% of the inoculated nude mice developed tumors in LDF-treated sample. Neither adherent cells nor nude mice developed tumors were found in M-LDF-treated samples.

**Discussion and Conclusion:**

Since M-LDF can effectively remove and destroy cancer cells in the salvaged blood, it has great potential for clinical application.

## Introduction

Surgical resection is still a preferred treatment for the solid tumors. One third of the banked blood is used during onco-surgery [1]. Unfortunately, blood transfusion may increase the risk of recurrence and mortality in patients with malignancies [2–4] with a shorter survival time [5]. Thus, an effective blood conservation strategy to minimize the adverse effects of allogeneic blood is needed during onco-surgery. Intraoperative blood salvage (IBS) has been used for 3 decades as an effective blood conservation strategy. However, it does not prevail in onco-surgery, because of malignant cell contamination in the salvaged blood [6]. Therefore, 50 Gy gamma irradiation has been applied to eliminate these malignant cells in Germany [7], but with limited use elsewhere [8]. A leukocyte depletion filter (LDF) can also remove tumor cells from the salvaged blood [9, 10]. Some clinical trials [11] suggest LDF used for IBS does not increase the risk of tumor recurrence, however, the potential metastasis still exists [12, 13], because not all nucleated cells can be removed. Therefore, its safety in application during onco-surgery is still questioned [1] and to further improve malignant cell removal is required. Our pilot studies indicate that pore size of the filter membrane and the wash solution are two key elements for cell depletion. Regular LDFs with a pore size of 25 μm remove 2–3 log of leukocytes from banked blood. However, salvaged blood, with the scarcity of plasma and platelets, is quite different from banked blood. Regular LDFs remove only 1–2 log of leukocytes and are ineffective for removal of malignant cells from the salvaged blood, which is consistent with another report [14]. With advances in materials and production processes, modified LDF (M-LDF) with a pore size as small as 12–18 μm is now available, which can remove 3–4 log of leukocytes in salvaged blood [15,16]. Mannitol-adenine-phosphate (MAP) solution not only helps preserve red blood cell (RBC) morphology and function and reduces hemolysis by improving energy production [17], but also improves tumor cell removal from 2–3 log to 4–5 log in IBS during onco-surgery [15,16]. In the present study, therefore, the effectiveness of this M-LDF on malignant cell removal was tested.

## Materials and Methods

Four protocols were approved by Institutional Ethics Review Boards of West China Hospital, Ethics Committee for Scientific Research and Clinical Trial of Medicine at Sichuan Cancer Hospital, and Human Subject Research Ethics Committee of Affiliated No. 2 Hospital of Zhejiang University, and carried out in West China Hospital (Protocols 1 and 2), Sichuan Cancer Hospital (Protocol 3) and Affiliated No. 2 Hospital of Zhejiang University (Protocol 4) respectively. Studies were conducted according to the Declaration of Helsinki. All patients signed informed consents before participation. Animal studies were approved by the Institutional Animal Ethical Committee at Affiliated No. 2 Hospital of Zhejiang University. Immunocompromised BALB/c *Foxn1* ^*nu*^ mice were from the Animal Center of Zhejiang University, and animals were euthanized by overdose of phenobarbital sodium at the end of the experiment. M-LDF was produced by Separator Haemo-Technology Beijing Co Ltd, Beijing, China, and regular LDF was produced by the Institute of Blood Transfusion, Chinese Academy of Medical Sciences. The salvaged blood was not transfused to patients in Protocol 2, 3 and 4. MAP solution (1.5g/L sodium citrate, 0.2g/L citric Acid, 7.93g/L glucose, 0.94g/L sodium biphosphate, 0.14g/L adenine, 4.97g/L sodium chloride, 14.57g/L mannitol) was produced by Sichuan Nigale Biomedical Co., Ltd., Chengdu, China.

### Protocol 1: Effect of M-LDF and LDF on removal of malignant cells in media

HepG2 cells (about 10^6^−10^7^) were harvested and mixed with 210 ml of MAP solution (n = 8). Ten ml of the suspension was sampled for cell count (1 ml), viability (4 ml) and culture (5 ml). The remaining was filtered with M-LDF, and cell count, viability (100 ml) and culture (100 ml) were assessed again. HepG2 cells were mixed with normal saline (NS 0.9 g/L sodium chloride) and filtered with a regular LDF as control (n = 8).

### Protocol 2: Effect of M-LDF on removal of malignant cells in shed blood

From April 1 to December 31, 2010, thirty patients (hepatic carcinoma or pancreatic cancer, Stage I) scheduled for tumorectomy were recruited. Shed blood was collected from the skin incision to tumor removal, as described before [15]. Blood was anti-coagulated (heparin 25 u/ml in MAP solution), and washed with an automated salvaging device (Jingjing Autologous Blood Recovery System, Model 3000P; Jingjing Medical Equipment Co., Ltd, Beijing, China) by MAP solution (1500–2000 ml for 250 ml RBC). Only salvaged blood, with hematocrit at 30–60% and greater than 250 ml of volume, was eligible for use.

Fourteen ml of the salvaged blood was sampled for cell count (2 ml), identification (4 ml) and culture (8 ml). The remaining blood was filtered with M-LDF, and reassessed for cell count (10 ml), identification (20 ml), and culture (40 ml). Filter membranes were also examined by light microscopy (BX51, Olympus, Japan) and transmission electron microscope (H-600IV, Hitachi, Osaka, Japan) for cell and filter interaction.

### Protocol 3: Performance of M-LDF on the tumor cell overload

From August to November, 2010, thirty patients (hepatic carcinoma, gallbladder cancer, bile duct cancer or pancreatic cancer, Stage I) were recruited in this protocol. Blood collection and washing procedures were the same as for Protocol 2. To mimic the overload of tumor cells in the salvaged blood, freshly removed tumors (about 2×2×2 cm^3^) were treated for single-tumor cell suspension [16]. Briefly, the tumor was mechanically and enzymatically dissected. The cells were isolated by centrifugation at 81× g for 3 min, and then suspended in phosphate-buffered saline (PBS). The isolated tumor cells were mixed with the salvaged blood and used for M-LDF assessment as described in Protocol 2.

### Protocol 4: Safety assessment of M-LDF

From August to November, 2010, a total of 30 patients were recruited in this protocol. Exclusion criteria, blood collection and washing procedure were the same as for Protocol 2. The HepG2 cell (about 10^6^−10^7^) at exponential growth phase was mixed with 250 ml of salvaged blood. Samples were taken for cell count, identification and culture as in Protocol 2, both before and after M-LDF treatment. In addition, nucleated cells were isolated from the salvaged blood (50ml before and 120 ml after M-LDF treatment, respectively), and injected subcutaneously into the left axilla of BALB/c *Foxn1* ^*nu*^ mice for bioassay.

#### Cellular viability

HepG2 cells were concentrated before and after LDF treatment in Protocol 1. Cellular viability was determined by flow cytometry analysis. Cells were incubated with 15*μ*M propidium iodide (PI, red, Sigma, USA) and 10 *μ*M calcein acetoxy-methyl ester (calcein-AM, green, Dojindo, Japan) for 5 min and 30 min respectively with no light exposure, and then washed by PBS. Data acquisition was performed on Calibur (BD-FACS Aria, BD, USA) with FACS Diva 5.0 software and analyzed by FCS Express (De Novo Software, Thornhill, ON). PI-negative and calcein-AM-positive cells were considered as viable. After filtration, HepG2 cells stained with PI and calcein-AM were also examined with a laser confocal microscope (Nikon A1, Nikon, Japan) for morphology.

#### Blood cell count and morphology

The RBC count was determined by an automated blood analyzer (MINDRAY, BC-3000, China). The salvaged blood was lysed with ammonium chloride solution at room temperature, and centrifuged at 300×g for 5 min. Nucleated cells were counted under a light microscope. Morphology and bare nuclei of nucleated cells were identified with Wright's stain [15, 16]. At least 100 cells per slide were counted.

To test the sensitivity of this cell separation method, 5 HepG2 cells labeled with AE1/AE3-PE (Anti-pan cytokeratin, clone AE1 /AE3, Cat: 53–9003, eBioscience, USA) were added into 50 ml of the salvaged blood. The blood was lysed, and nucleated cells were isolated (see above) and smeared on a slide. Labeled cells were counted under a fluorescence microscope (DM4000B Leica- Ernst-Leitz, St. Wetzlar, Germany). After repeating counts 5 times, 24 cells in total of 25 cells (96%) were found in 5 samples.

#### Identification of nucleated cells

Nucleated cells obtained after erythrolysis were smeared on glass slides, air dried, and fixed in 4% paraformaldehyde. To identify malignant cells, slides were stained with CK19-PE (Cat# bsF-1028R, Beijing Bioss Biotechnology Co., Ltd., Beijing, China) in protocol 2 [18], and with AE1/AE3-PE in protocols 3 and 4 [15,16]. To further identify clones or invasive growth cells in hepatoma samples, CD133-FITC (Cat: 12-1338-42, eBioscience, USA) was selected in protocols 2 and 3 (n = 7 in each) [15, 16, 19–21]. The glass slides were put into a dark-box with CK19-PE or AE1/AE3-PE overnight at 4°C, followed by PBS washing. Then DAPI was applied for 10 min, and examined under a fluorescence microscope.

#### Cell culture and identification

For cell culture, the nucleated cells were isolated by centrifugation as in Protocol 1, and by density gradient centrifugation as in protocols 2, 3 and 4 [15, 16]. The salvaged blood was diluted 1:1 with sterile PBS. Then lymphocyte separation medium (LTS1077-1 TBD, China) was slowly added to the blood, and centrifuged at 300×g for 15 min at room temperature. The intermediate white layer was extracted and washed 3 times in PBS to isolate nucleated cells. The cells were cultured in the glucose-rich DMEM media (SH30022, Hyclone, USA) supplemented with 10% fetal bovine serum (SV30087.02 Hyclone, USA) at 37°C. The medium was refreshed every 24 hrs. After 15 days of incubation, the medium was removed, and the cells were fixed in 4% paraformaldehyde for 10 min, and stained with CK19-PE or AE1/AE3-PE in the last 3 protocols.

To assess proliferative capacity of malignant cells, nucleated cells from samples before (×10^7^ cells) and after M-LDF treatment (×10^3^ cells) in protocol 4 were injected respectively into the left axilla of immunocompromised BALB/c *Foxn1* ^*nu*^ mice (4 week old, n = 60). Tumors that were 2–3 cm in diameter at inoculation site were removed for pathological and immunofluorescence examination. If no tumor was observed after one year, tissues at injection sites were then obtained and examined.

### Statistical analysis

All data were analyzed by SPSS18.0 (Chicago, IL). All the quantitative data were examined for their distribution. Normal distribution data were expressed by the mean ± standard deviation, and one-way ANOVA was used to compare the difference before and after treatment. Non-normal distribution data were expressed by a median (min, max) and the Kruskal-Wallis test was applied to compare differences among groups. Rates of tumor development in nude mice were expressed as a percentage, and examined with the Fisher exact test (protocol 4). P<0.05 was considered statistically significant.

## Results

### Protocol 1: M-LDF was more effective than regular LDF in cell removal

**Fig 1:**
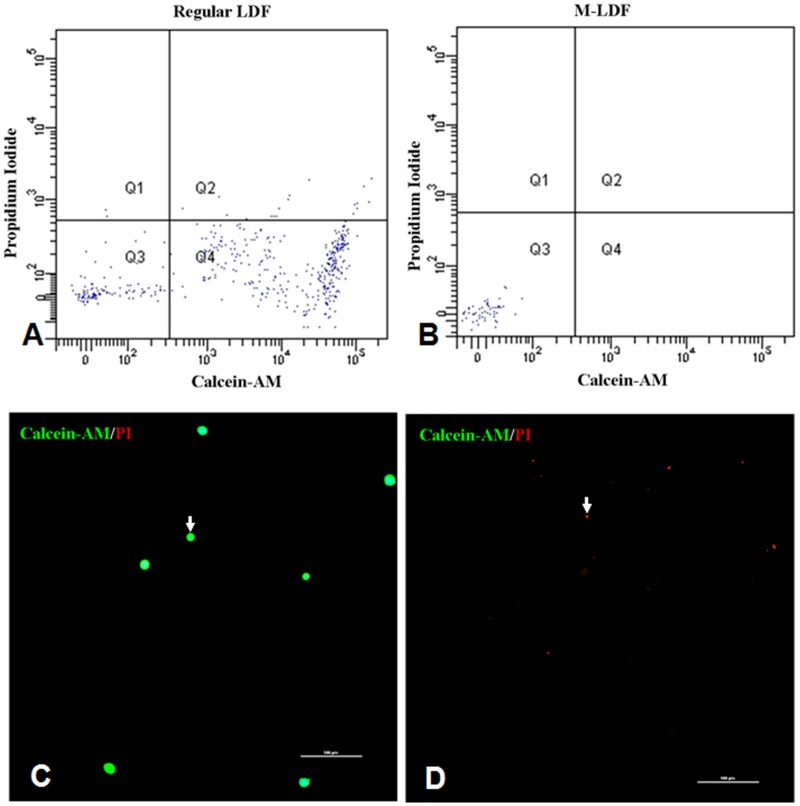
Flow cytometry and confocal microscopy for identification of viable HepG2 cells. Cells treated with LDF or M-LDF were stained with PI and calcein-AM for viability assessment. Viable cells were found in the LDF group (white arrow, A and C), but not in the M-LDF group (B and D).

HepG2 cell count was (7.55±0.63)×10^6^ cells/200 ml before filtration. The count decreased to (2±4) cells/200 ml in the M-LDF group, and to (1.39±2.90) ×10^3^ cells/200 ml in the LDF group. No viable cells or cell colonies were found in M-LDF-treated samples, except fragmented debris (Fig 1), suggesting cells were damaged when they passed through the filter. However, both viable cells (100%) (Fig 1) and cell colonies (37.5%)(S1 Fig) were found in LDF samples.

### Protocol 2: Effect of M-LDF on removal of tumor cells

**Fig 2:**
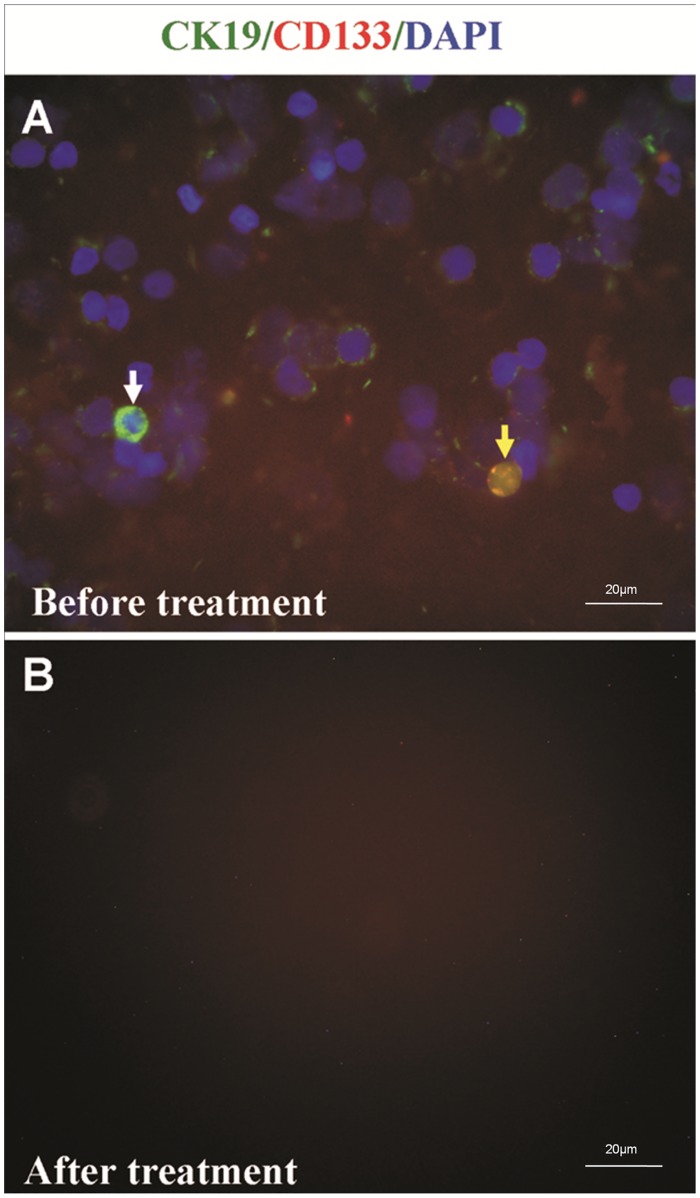
Immunofluorescence staining for detection of malignant cells in the salvaged blood. Blood was collected and washed with an automated salvaging device. Nucleated cells were stained with CK19-PE (white arrow) and CD133-FITC (yellow arrow) (A). Positive cells were eliminated after M-LDF treatment (B).

**Fig 3:**
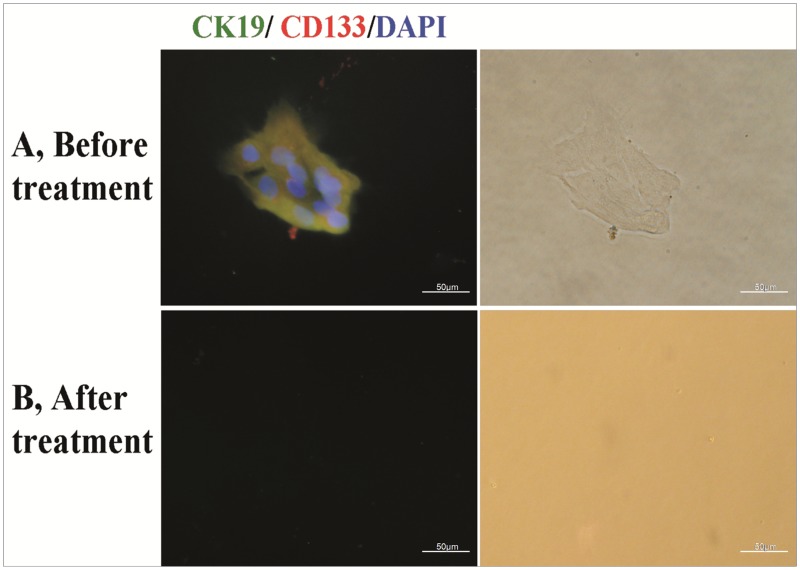
Identification of adherent cells. Nucleated cells were isolated by density gradient centrifugation for cell culture. Adherent cells were identified as in Fig 2. A: Adherent cells and/or clones (CK19^+^ and CD133^+^) were found in 65% of samples before (A), but none after the M-LDF treatment (B).

**Fig 4:**
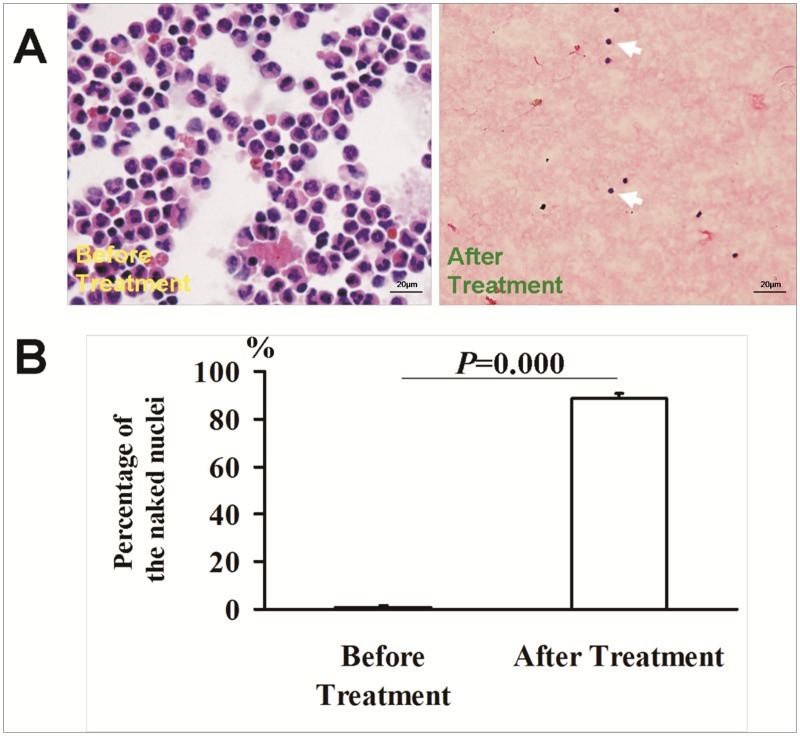
Morphology of the nucleated cells in blood before and after M-LDF treatment. A light microscopic view of nucleated cells (Wright's stain) (A). Naked cells were identified as blue and without circular red staining (white arrow). About 90% of cells were naked nuclei after M-LDF treatment vs 1% before the treatment (B).

**Fig 5:**
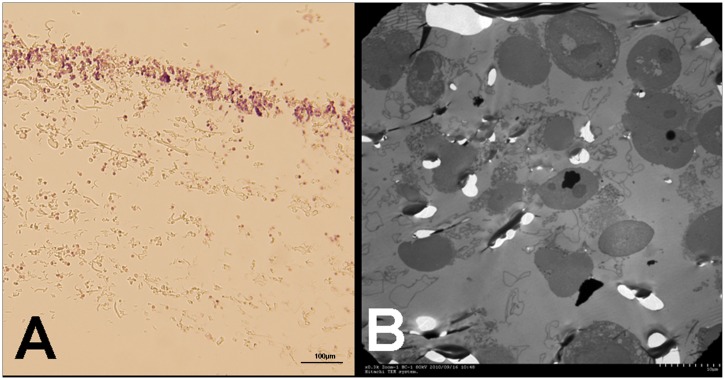
Ultrastructure of the M-LDF membrane. After filtration, the first layer membrane of M-LDF was examined by light microscopy (A) and transmission electron microscopy (B). A: Nucleated cells were mostly trapped in the superior 1/3 part of the membrane. B: The trapped cells were ruptured with cytoplasm leakage.

There were 30 patients in Protocol 2 (Table 1). Nucleated cell counts in salvaged blood decreased from (3.34±1.28)×10^9^/L of baseline to (5.17±3.78) ×10^5^/L after M-LDF treatment (P = 0.000), while 96% of RBCs were recovered. M-LDF filtration eliminated both CK19^+^ and CK19^+^CD133^+^ cells (Fig 2). The adherent cells, CK19^+^ and CD133^+^, were characterized by invasive growth in 70% of untreated samples. They were also eliminated by M-LDF filtration (n = 7, Fig 3). Cells that passed through the M-LDF became naked (Fig 4). Ultrastructure of the M-LDF membrane showed ruptured cell membranes and cytoplasm leakage (Fig 5).

**Table 1:** Patient characteristics in Protocols 2 and 3.

	Value
**Protocol 2 (n = 30)**	
Gender (male/female)	23/7
Age (year, mean±SD)	54±9
Body mass (kg)	66±11
**Diagnosis**	
Hepatoma	24
Pancreatic cancer	6
Tumor grade (high/medium/low)	2/9/19
Tumor size (cm^3^)	131 (0.21–533)
**Protocol 3 (n = 30)**	
Gender (male/female)	25/5
Age (year, mean±SD)	52±13
Body mass (kg)	62±10
**Diagnosis**	
Hepatoma	25
Gallbladder cancer	2
Bile duct cancer	2
Pancreatic cancer	1
Tumor grade (high/medium/low)	2/12/16
Tumor cells added (×10^7^)	8.74 (0.26–294.00)

### Protocol 3: Performance of M-LDF during tumor cell overload

**Fig 6:**
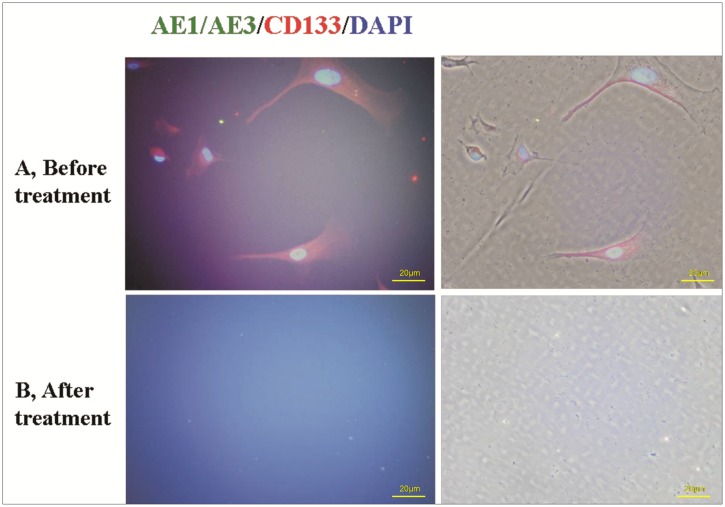
Identification of adherent cells with immunofluorescence staining. Cell suspensions were prepared by adding 8.74×10^7^ tumor cells from the same patient to the salvaged blood. Adherent cells were stained with AE1/AE3-PE and CD133-FITC. Adherent cells (both AE1/AE3^+^ and CD133^+^) that were characterized by invasive growth were found in 85% of samples (A), but none after the M-LDF treatment (B).

The filtrate contained as high as 8.74×10^7^ tumor cells (range from 0.26 to 294.00 ×10^7^) / 250 ml of salvaged blood, M-LDF removed up to 4 log of nucleated cells. AE1/AE3^+^ cells were found in all untreated-samples, and invasive growth cells were found in 85% of untreated-samples (n = 7, Fig 6). They were absent after M-LDF filtration.

### Protocol 4: Safety assessment of M-LDF

**Fig 7:**
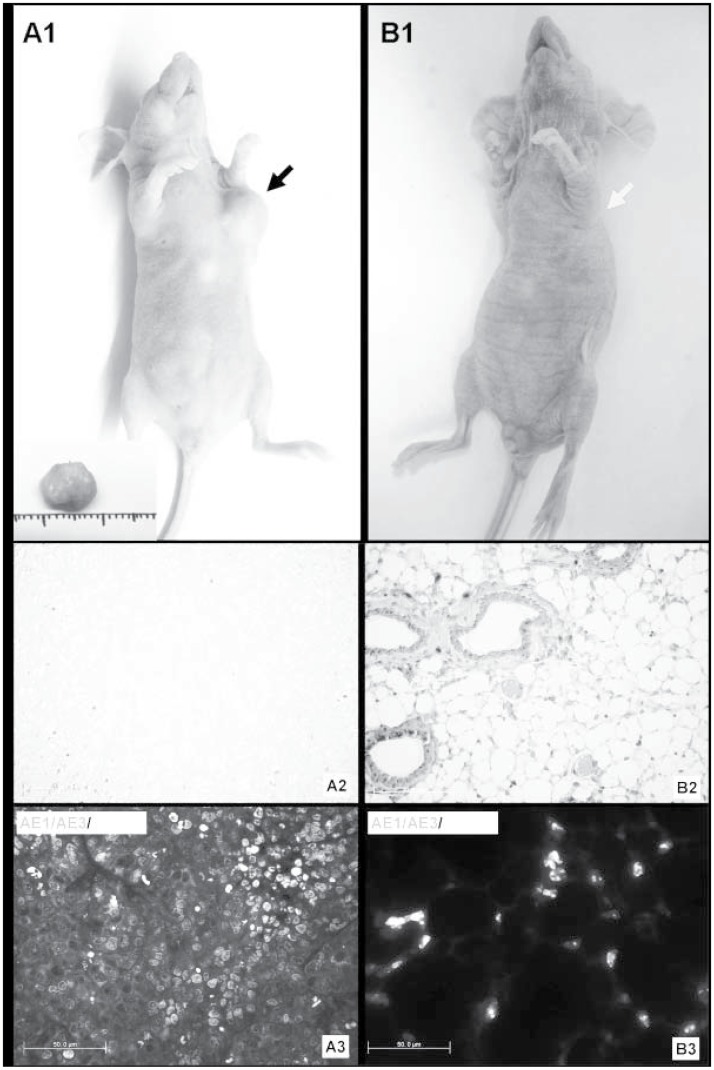
Bioassays for tumor development in nude mice. Injection of M-LDF untreated and treated samples into the left axilla of nude mice. With untreated samples, tumors developed (2–3 cm in diameter, A1, Black arrow) and were removed for HE (A2) and immunofluorescent staining (AE1/AE3-PE and DAPI, A3) to confirm human origin. No tumor was found after one year (B1, green arrow) in the M-LDF treated samples. HE staining of tissues from the axilla showed normal cells (B2), and stained negative for AE1/AE3 (B3).

M-LDF filtration reduced nucleated cell count from (2.46±1.63) ×10^9^/L to (4.54±12.04) ×10^4^/L (P = 0.000). With unfiltered samples, clones were found in all cultures within 3–5 days, and tumors developed in 20 out of 30 inoculated nude mice (72 days, ranging from 5 to 338 days, Fig 7A1), which was confirmed by pathological and immunofluorescent examinations (Fig 7A2 and 7A3). Neither clones were found in culture and nor tumors developed in mice in the M-LDF-treated group (Fig 7B).

## Discussion and Conclusion

For the first time we demonstrated that M-LDF is very effective in removing cells. It removed 4 log of nucleated cells and more malignant cells (protocol 1), with 96% RBC recovery. Furthermore, nucleated cells were destroyed as they passed through the M-LDF. No viable proliferative or epithelial cells were found after M-LDF filtration. Therefore, M-LDF may have potential for use during onco-surgery.

As reported, cancer cells were in salvaged blood [6] and regular LDF can only remove 2–3 log of malignant cells. Our results confirmed the previous studies [14, 22]. Therefore, there is a risk of metastasis for using salvaged blood [23] and it is not safe for onco-surgery patients. Interestingly, meta-analysis [24] plus a clinical trial [25] show that IBS decreased the risk of metastasis. In our study, we found cancer cells and invasive growth cells in salvaged blood (Protocol 3), which caused malignant tumors in 67% of nude mice (Protocol 4). This discrepancy may result for two reasons. First, the seed requires the right microenvironment at the right time for survival [26]. Therefore, less than 0.01% of seeds formed metastatic lesions [26]. Second, invasive growth cells in hepatoma samples were identified as CD133^+^, suggesting metastasis is likely caused by a specific subtype of cancer cells, which are scarce in the salvaged blood.

Whether malignant cells in IBS will affect prognosis remains unknown, however, it is prudent to remove malignant cells before transfusion. Our results show that the M-LDF is effective in eliminating the malignant cells (5 log) even with cell overloading (10^8^/L). We did not find viable malignant cells by flow cytometry, immunofluorescence or cell culture in vitro and in vivo after M-LDF filtration as reported [11, 14, 15, 16]. Cells may be destroyed or lose their adhesive characteristics, which is critical for metastasis formation, as they pass through the filter.

In conclusion, our results demonstrate that the M-LDF can effectively remove a variety of cells. Since no viable malignant cells were found in salvaged blood after M-LDF filtration, IBS has significant potential for clinical application in onco-surgery.

## Supporting Information

S1 Fig Identification of proliferative cells in cell culture. All precipitate separated by centrifugation after treatment with regular LDF or M-LDF was used for a 15 day cell culture. A: Clones were found in 3 out of the 8 regular LDF treated blood samples. B: No clones were detected in the M-LDF treated samples.
(EPS)
